# Yokukansan Inhibits Neuronal Death during ER Stress by Regulating the Unfolded Protein Response

**DOI:** 10.1371/journal.pone.0013280

**Published:** 2010-10-12

**Authors:** Toru Hiratsuka, Shinsuke Matsuzaki, Shingo Miyata, Mitsuhiro Kinoshita, Kazuaki Kakehi, Shinji Nishida, Taiichi Katayama, Masaya Tohyama

**Affiliations:** 1 Department of Anatomy and Neuroscience, Graduate School of Medicine, Osaka University, Suita, Japan; 2 Department of Child Development and Molecular Brain Science, United Graduate School of Child Development, Osaka University, Kanazawa University and Hamamatsu University School of Medicine, Suita, Japan; 3 The Osaka-Hamamatsu Joint Research Center for Child Mental Development, Graduate School of Medicine, Osaka University, Suita, Japan; 4 Laboratory of Biopharmaco Informatics, School of Pharmaceutical Sciences, Kinki University, Higashiosaka, Japan; 5 Department of Kampo Medicine, Graduate School of Medicine, Osaka University, Suita, Japan; RIKEN Brain Science Institution, Japan

## Abstract

**Background:**

Recently, several studies have reported Yokukansan (Tsumura TJ-54), a traditional Japanese medicine, as a potential new drug for the treatment of Alzheimer's disease (AD). Endoplasmic reticulum (ER) stress is known to play an important role in the pathogenesis of AD, particularly in neuronal death. Therefore, we examined the effect of Yokukansan on ER stress-induced neurotoxicity and on familial AD-linked presenilin-1 mutation-associated cell death.

**Methods:**

We employed the WST-1 assay and monitored morphological changes to evaluate cell viability following Yokukansan treatment or treatment with its components. Western blotting and PCR were used to observe the expression levels of GRP78/BiP, caspase-4 and C/EBP homologous protein.

**Results:**

Yokukansan inhibited neuronal death during ER stress, with Cnidii Rhizoma (Senkyu), a component of Yokukansan, being particularly effective. We also showed that Yokukansan and Senkyu affect the unfolded protein response following ER stress and that these drugs inhibit the activation of caspase-4, resulting in the inhibition of ER stress-induced neuronal death. Furthermore, we found that the protective effect of Yokukansan and Senkyu against ER stress could be attributed to the ferulic acid content of these two drugs.

**Conclusions:**

Our results indicate that Yokukansan, Senkyu and ferulic acid are protective against ER stress-induced neuronal cell death and may provide a possible new treatment for AD.

## Introduction

Yokukansan (Tsumura TJ-54), a traditional Japanese medicine, has traditionally been administered to patients who show symptoms such as nervousness, short-temperedness, irritability, sleeplessness, twitching of the eyelids and shaking of the limbs. It has also been administered to infants who suffer from night crying, restlessness and convulsions. Recently, several clinical reports have shown that Yokukansan is effective against the Behavioral and Psychological Symptoms of Dementia (BPSD) and improves daily living of patients [1]–[3]. Thus, Yokukansan has been suggested as a possible new candidate for treating Alzheimer's disease (AD). However, no basic research on the clinical effects of Yokukansan has been conducted.

Many reports have suggested that endoplasmic reticulum (ER) stress is involved in the pathogenesis of AD, with several studies showing that the amyloid β protein, which is abundant in the AD brain, induces ER stress [4]–[6]. Previous studies from our laboratory have shown that the familial AD (FAD)-linked presenilin-1 (PS1) mutation increases the susceptibility to ER stress and that the presenilin-2 (PS2) splice variant (PS2V), observed in the sporadic form of AD, also increases the risk of ER stress [7]–[12]. These results suggest that ER stress is involved in the pathogenesis of AD.

ER stress activates both the survival and apoptotic pathways. In the survival pathway, ER stress induces the transcription of genes encoding for the ER-resident chaperones such as GRP78/Bip, GRP94 and protein disulfide isomerase (PDI), which facilitate protein folding. This induction system is termed the ‘unfolded-protein response (UPR) [13]–[16]. By contrast, the representative gene C/EBP homologous protein (CHOP), also known as growth arrest and DNA damage-inducible gene 153 (GADD153), is induced in the apoptotic pathway [16]–[17]. In addition, we have revealed the involvement of caspase-4, a protease that is specifically induced by ER stress in humans and may be involved in the pathogenesis of AD [18]. The familial AD-linked PS1 mutation accelerates the cleavage of caspase-4, which in turn activates caspase-3 and caspase-9 without involving the cytochrome-c pathway [19]. These results suggest that the initiation of caspase-4 cleavage is one of the key events for the pathogenesis of AD.

In this report, we studied the effect of Yokukansan on ER stress-induced neurotoxicity and on FAD-linked PS1 mutation (ΔE9) associated cell death. We determined that upregulation of GRP78/Bip expression by Yokukansan, as well as the inhibition of CHOP induction, results in a reduction of ER stress-induced cell death and FAD-linked associated cell death. In addition, we showed that Yokukansan inhibits the activation of caspase-4. Furthermore, we exhibited that the effects of Yokukansan could be attributed to the function of Cnidii Rhizoma (Senkyu), a component of Yokukansan. We determined that the ferulic acid contained in Senkyu plays an important role for the protective function of Yokukansan or Senkyu. These results show that Yokukansan, Senkyu or ferulic acid alone could be a potential treatment for AD and our findings cast new light on the development of new therapies for AD.

## Results

### Yokukansan reduces ER stress-induced neuronal cell death

**Figure 1:**
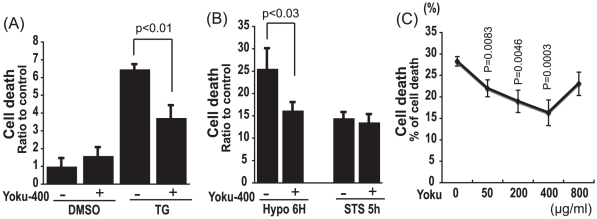
Yokukansan reduces ER stress-induced neuronal cell death. Cell toxicity in N_2_a cells was measured based on morphological changes. Quantitative data are expressed as the mean ± SEM for at least three independent experiments. The P value was compared with the control and calculated by Student's T test. (A) Cell death was measured 6.5 h after 1 µM TG or DMSO (control) exposure with or without 1.5 h of pretreatment with 400 µg/ml Yokukansan. (B) Cell death was measured 6 h after hypoxia exposure and 5 h after 0.1 µM STS exposure with or without 1.5 h of pretreatment with 400 µg/ml Yokukansan. Non treated cells were used as the control. (C) Cell death was measured 6.5 h after 1 µM TG exposure with 1.5 h of pretreatment with the indicated concentration of Yokukansan.

We examined the effects of Yokukansan on neuronal cell death caused by several stresses using the mouse neuroblastoma cell line, Neuro2a (N_2_a). Thapsigargin (TG) and hypoxia were used as ER stress inducers and staurosporine (STS) was used as a mitochondrial stress inducer. Yokukansan significantly decreased the cell death caused by TG and hypoxia (Figure 1A and 1B), but did not protect against STS treatment (Figure 1B). These results indicate that Yokukansan is effective against ER stress-induced neuronal toxicity that involves impairment of calcium homeostasis, but not apoptotic stimuli that do not cause ER stress. Notably, as shown in Figure 1C, the protective effect of Yokukansan against ER stress-induced cell death is proportional to the concentration of Yokukansan used. However, a high dose of Yokukansan showed some toxicity.

### Cnidii Rhizoma (Senkyu), a component of Yokukansan, has a potent protective effect against ER stress-induced neuronal toxicity

**Figure 2:**
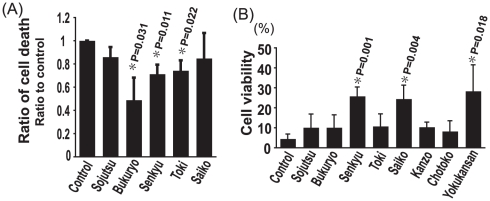
Cnidii Rhizoma (Senkyu), a component of Yokukansan, has potent protective effects against ER stress- induced neuronal toxicity. N_2_a cell toxicity and SK-N-SH cell viability was measured based on morphological changes and the WST-1 assay, respectively. Quantitative data are expressed as the mean ± SEM for at least three independent experiments. The P value was compared with the control and calculated by Student's T test. (A) Cell death was measured 20 h after 1 µM TG exposure with 1.5 h pretreatment with 200 µg/ml of each of the indicated components of Yokukansan. (B) Cell viability was measured 3 h after 3 µM TG exposure with 1.5 h pretreatment with 200 µg/ml of each of the indicated components of Yokukansan. Cells incubated with TG without any pretreatment were used as a control.

To determine which component of Yokukansan plays a key role in inducing the protective effect against neuronal cell death caused by ER stress, we examined the effect of each of the 7 components of Yokukansan on neuronal death caused by TG using the N_2_a cell line. Cnidii Rhizoma (Senkyu), Hoelen (Bukuryo) and Angelicae Radix (Toki) significantly decreased cell death caused by TG, and Bupleuri Radix (Saiko) showed some neuroprotective effect against TG (P = 0.054) (Figure 2A). However, the other components of Yokukansan (Atractylodis Lanceae Rhizoma (Soujutsu), Glycyrrhizae Radix (Kanzo) and Uncariae Uncis Cum Ramulus (Chotoko)) failed to inhibit cell death caused by TG (Figure 2A and data not shown). When using the human neuroblastoma cell line SK-N-SH, Senkyu and Saiko induced a significant reduction in neuronal death caused by TG (Figure 2B). However, the other components of Yokukansan did not show any neuroprotective effect against TG. Senkyu was the most potent inhibitor of neuronal cell death following TG-induced toxicity in both N_2_a and SK-N-SH cells. Therefore, our subsequent analysis focused on the effect of Senkyu on neuronal death caused by ER stress.

### Senkyu and Yokukansan reduce neuronal toxicity caused by the FAD-linked PS1 mutation

**Figure 3:**
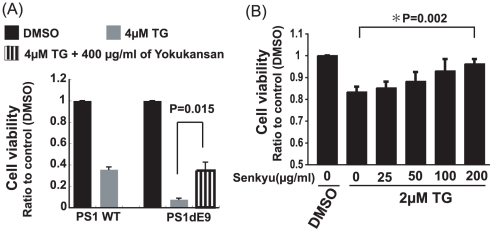
Senkyu and Yokukansan reduce neuronal toxicity caused by the FAD linked PS1 mutation. Cell viability of SK-N-SH cells expressing PS1 WT or PS1ΔE9 was measured using the WST-1 assay. Quantitative data are expressed as the mean ± SEM for at least three independent experiments. The P value was compared with the control (DMSO treated cells) and calculated by Student's T test. (A) Cell viability was measured 3 h after 4 µM TG exposure with or without a 1.5 h pretreatment with 400 µg/ml of Yokukansan. (B) Cell viability of cells expressing PS1ΔE9 was measured 3 h after 2 µM TG exposure with a 1.5 h pretreatment with Senkyu at the indicated concentrations (0, 25, 50, 100, 200 µg/ml).

Previously, we demonstrated that mutations in PS1 increase vulnerability to ER stress by altering the signaling pathway. We stably transfected SK-N-SH cells with complementary DNA constructs encoding for wild-type PS1 and PS1 with a deletion of exon 9 (ΔE9), which is one of the FAD-linked mutations. As shown in Figure 3A, the addition of TG induced a greater cell death rate in cells expressing the mutant PS1 when compared with cells expressing the wild-type protein. In addition, pretreatment with Yokukansan reduced cell death to wild type levels in TG treated cells expressing ΔE9. Moreover, Senkyu inhibited cell toxicity following TG treatment in a concentration-dependent manner, resulting in levels similar to those seen in dimethyl sulfoxide (DMSO)-treated cells (Figure 3B). These results indicate that Yokukansan may be able to rescue cells from ER stress caused by the AD-linked mutation through the effect of Senkyu.

### Senkyu and Yokukansan reduce the vulnerability to ER stress by altering the unfolded protein response (UPR) signaling pathway

**Figure 4:**
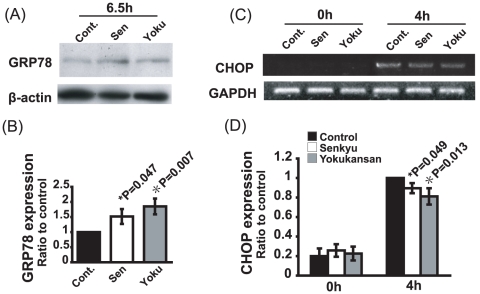
Senkyu and Yokukansan reduce susceptibility to ER stress by altering the unfolded protein response (UPR) signaling pathway. (A and B) SK-N-SH cells were treated with Senkyu or Yokukansan (200 µg/ml) for 6.5 h. Cells were lysed and western blot analysis was performed using an anti-Bip or anti-b-actin primary antibody (A). Quantitative data were obtained by densitometry of the bands. Data are expressed as the mean ± SEM for at least three independent experiments (shown as a ratio of the control). The P value was compared with the control and calculated by Student's T test (B). (C and D) SK-N-SH cells were treated with 1 µM TG with or without a 1.5 h pretreatment with Senkyu or Yokukansan (200 µg/ml). The expression of CHOP mRNA and GAPDH mRNA were detected by RT-PCR (C). Quantitative data were obtained by densitometry of the bands. Data are expressed as the mean ± SEM for at least three independent experiments (shown as a ratio of the control). The P value was compared with the control and calculated by Student's T test (D).

Normal cells respond to ER stress by increasing the transcription of genes encoding for the ER-resident chaperons such as GRP78/Bip, GRP94 and PDI, which facilitate protein folding (unfolded protein response). An increase in GRP78/Bip expression leads to cell survival. However, ER stress can also induce CHOP expression and activation of the JNK pathway, which induce cell death. Therefore, to determine the molecular mechanism of the neuroprotective effect of Senkyu and Yokukansan against ER stress, we examined the basal expression levels of GRP78/Bip and the expression levels of mRNA encoding for CHOP following TG toxicity after Senkyu or Yokukansan treatment. Both Senkyu and Yokukansan upregulated GRP78/Bip expression when compared with the no treatment control (Figures 4A and 4B). By contrast, both Senkyu and Yokukansan treatment significantly reduced CHOP expression when compared with cells treated with TG alone (Figures 4C and 4D).

### Senkyu and Yokukansan inhibit the activation of caspase-4, an ER stress-specific apoptotic protease

**Figure 5:**
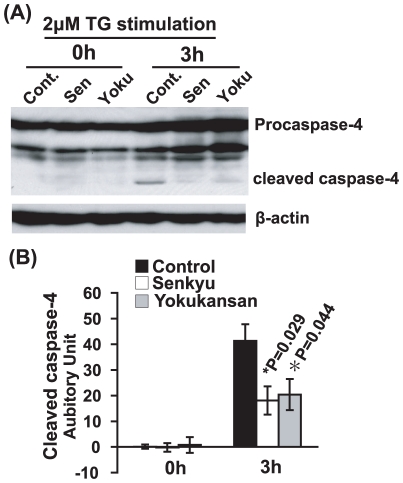
Senkyu and Yokukansan inhibit the activation of caspase-4. (A and B) SK-N-SH cells were treated with 2 µM TG for 3 h with or without a 1.5 h pretreatment with Senkyu (200 µg/ml) or Yokukansan (200 µg/ml). Cells were lysed and western blot analysis was performed using an anti-caspase-4/TX or anti-b-actin antibody (A). Quantitative data were obtained by densitometry of the bands. Data are expressed as the mean ± SEM for at least three independent experiments (shown as a ratio of the control). The P value was compared with the control and calculated by Student's T test (B).

**Figure 6:**
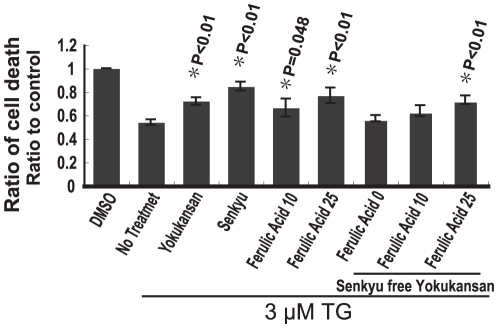
Ferulic acid, a component of Cnidii Rhizoma (Senkyu), has potent protective effects against ER stress-induced neuronal toxicity. Cell viability of SK-N-SH cells was measured using the WST-1 assay. Quantitative data are expressed as the mean ± SEM for at least three independent experiments. The P value was compared with the control (DMSO treated cell) and calculated by Student's T test. Cell viability was measured 3 h after 3 µM TG exposure with or without a 2 h pretreatment with 200 µg/ml of Yokukansan, Senkyu-free Yokukansan or Senkyu, with or without ferulic acid at the indicated concentrations (0, 10, 25 µg/ml). Cells incubated with TG without any pretreatment were used as a control.

Caspase-4 has been shown to be involved in ER stress-induced neuronal cell death and in the pathogenesis of AD [18], [19]. We have shown that Yokukansan and Senkyu reduce ER stress-induced neuronal cell death (Figures 1, 2, 3 and 6). Therefore, we examined the effect of Yokukansan and Senkyu on the activation of caspase-4. As shown in Figures 5A and 5B, both Senkyu and Yokukansan inhibited the cleavage of caspase-4. Thus, the protective effect of Yokukansan and Senkyu against ER stress can be partially attributed to the inactivation of caspase-4 as well as regulation of the UPR.

### Ferulic acid plays an important role in cell survival during ER stress

Our results indicate that Yokukansan induces resistance against ER stress by regulating the UPR and apoptotic pathway, particularly following Senkyu treatment, one of the components of Yokukansan. To confirm our results, we examined the effect of Senkyu-free Yokukansan, which contains all components except Senkyu. The Senkyu-free Yokukansan did not improve cell viability (Figure 6). These results show that Senkyu is important for survival during ER stress-induced toxicity. To determine how Senkyu induces its neuroprotective effect, we screened the contents of Senkyu as shown in Figure S1. As a result, we identified two potent candidates, ferulic acid and coniferyl ferulate (Figure S2). Ferulic acid, a plant constituent, has been reported to be a strong free radical scavenger with an antioxidant capacity [20]. In addition, ferulic acid has many pharmacological effects such as anti-inflammatory, anticancer, anti-diabetic, anti-atherogenic and neuroprotective [21]–[25]. Furthermore, ferulic acid has been reported to be protective against amyloid β protein toxicity [26], [27]. Thus, we focused on ferulic acid. To elucidate the function of ferulic acid, we monitored the effect of ferulic acid pretreatment on cell viability following ER stress. Ferulic acid was neuroprotective against ER stress in a concentration-dependent manner and provided similar protection to that of Yokukansan- or Senkyu-pretreated cells (Figure 6). In addition, we confirmed this result by treating cells with a mixture of ferulic acid and Senkyu free Yokukansan (Figure 6).

### Effect of Yokukansan, Senkyu and Ferulic acid on the UPR

**Figure 7:**
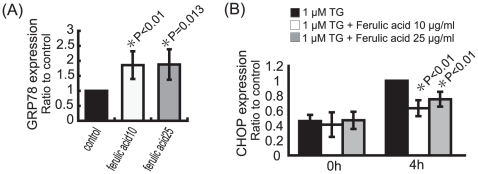
Ferulic acid, Senkyu and Yokukansan reduce susceptibility to ER stress by altering the unfolded protein response (UPR) signaling pathway. (A) SK-N-SH cells were treated with ferulic acid at the indicated concentrations (0, 10, 25 µg/ml) for 2 h. (B) SK-N-SH cells were treated with 1 µM TG with or without a 2 h pretreatment with ferulic acid at the indicated concentrations (0, 10, 25 µg/ml). (A, B) The expression of GRP78/Bip (A), CHOP (B) and GAPDH (A, B) mRNA were detected by RT-PCR. Quantitative data were obtained by densitometry of the bands. Data are expressed as the mean ± SEM for at least three independent experiments (shown as a ratio of the control). The P value was compared with the control and calculated by Student's T test.

We have shown that induction of GRP78/Bip and reduction of CHOP following pretreatment with Yokukansan or Senkyu reduces cell toxicity caused by ER stress. Therefore, we examined the effect of ferulic acid on the expression levels of GRP78/Bip and CHOP. Pretreatment with ferulic acid increased the mRNA expression level of GRP78/Bip. A similar result was observed following pretreatment with Yokukansan or Senkyu (Figures 4A, B and 7A). In addition, CHOP induction, caused by ER stress, was reduced following pretreatment with ferulic acid, as was seen following pretreatment with Yokukansan or Senkyu (Figure 4C, D and 7B). These results suggest the involvement of ferulic acid in the regulation of the UPR signaling pathway.

## Discussion

Recently, several clinicians have observed the effectiveness of Yokukansan, a traditional Japanese medicine, in the treatment of the BPSD and in cognitive impairment of AD [1]–[3]. However, the molecular mechanism remains unclear. In this study, we examined the effect of Yokukansan on cell death caused by TG, STS or hypoxia. Yokukansan reduced the cell death caused by TG and hypoxia, both of which induce ER stress via abnormal Ca^2+^ homeostasis, but were unable to protect against neural toxicity caused by STS, a mitochondrial stress inducer. These results suggest that Yokukansan may not be effective against mitochondrial stress related toxicity, but on ER stress related cell toxicity (Figure 1). In addition, recent studies have reported that Yokukansan has preventive or inhibitive effects against the development of memory disturbance, BPSD-like behaviors and neurodegeneration, all of which are observed in thiamine deficient rodents because of ER stress due to thiamine deficicency [28]–[30]. These reports support our hypothesis that Yokukansan may play an important role against ER stress.

Yokukansan consists of several components. Therefore, it was important to determine which components were effective against ER stress. Thus, we investigated the effect of each component on TG induced cell death. As shown in Figure 2, Bukuryo and Toki reduced cell death in N_2_a cells, but not in SK-N-SH cells; Saiko inhibited cell death in SK-N-SH cells, but not that of N_2_a cells; only Senkyu rescued both N_2_a and SK-N-SH cells from TG-induced cell toxicity. Such differences in effects on the cell death observed following Bukuryo, Toki and Saiko pretreatment may be due to differences in the exposure time and concentration of TG or differences between the cell lines. Nevertheless, we focused on Senkyu because it was the only component that reduced cell death under both conditions.

As mentioned previously, our results provide strong evidence that Senkyu, a component of Yokukansan, protects against ER stress-induced neuronal cell death, particularly against ER-stress caused by intracellular calcium homeostasis abnormalities (Figure 1, 2, 3 and 6). In addition, pretreatment with Senkyu upregulated GRP78/Bip expression and down-regulated CHOP expression caused by ER stress (Figure 4). GRP78/Bip is known to protect cells from cell death caused by ER stress [13]–[16], while CHOP induces cell death during ER stress [16], [17]. Thus, Senkyu may inhibit ER stress-induced neuronal cell death via regulation of the UPR and the apoptotic cascade. We also examined the effect of Senkyu and Yokukansan on neuronal cell death caused by the down-regulation of the UPR signaling pathway as a result of FAD-linked PS1 mutations [7], [8]. As shown in Figure 3, both Yokukansan and Senkyu improved the viability of cells under TG stimulation. In addition, we elucidated that Yokukansan and Senkyu inhibited the activation of caspase-4 observed under ER stress (Figure 5). Thus, the reduction of ER stress-induced cell death could be attributed to the inactivation of caspase-4 and regulation of the UPR signaling pathway. These findings suggest that Yokukansan or Senkyu alone may be an effective candidate for the treatment of AD.

Based on these findings, we proceeded to screen the contents of Senkyu and Yokukansan to determine their function. As a result, we found ferulic acid, which has been shown to be protective against amyloid β toxicity and oxidative stress [26], [27]. As shown in Figure 6, similar to Yokukansan and Senkyu, ferulic acid reduced cell death following ER stress. Furthermore, ferulic acid regulated the expression levels of GRP78/Bip and CHOP (Figure 7). These findings indicate that the protective effects of Yokukansan and Senkyu are due to ferulic acid.

We also observed that long term treatment or a high dose of Yokukansan had a neurotoxic effect on cultured neuronal cells (Figure 1C) and that some components of Yokukansan (Kanzo and Chotoko) also had the same effect with respect to neurotoxicity (Figure S3). However, longer exposure to Senkyu or ferulic acid did not induce neurotoxicity (data not shown). These data indicate that Senkyu or ferulic acid would be more clinically advantageous in terms of safety.

Several studies have reported that Chotosan, another traditional Japanese medicine, is also effective against amyloid β toxicity [31]–[33]. Similar to Yokukansan, the components of Chotosan, which include Chotoko, Bukuryo and Kanzo, activate neprilysin and insulin degrading enzyme (IDE), both of which are proteases of the amyloid β protein [33]. Given that Chotosan does not contain Senkyu, it is possible that some of the neuroprotective effects seen with Yokukansan following amyloid β toxicity could be attributed to the other components of Yokukansan. However, the presence of ferulic acid in Yokukansan does partially explain the neuroprotective effect observed following amyloid β protein toxicity [26]–[27]. Considering the following results: 1: A high dose of Yokukansan causes neural toxicity, but a high dose of Senkyu or ferulic acid does not show any toxicity (Figures 1C and 3B, Figure S3 and data not shown), 2: Senkyu reduces cell death following PS1 mutations [7], [8] (Figure 3), 3: Senkyu and ferulic acid reduce cell death caused by Ca^2+^-related ER stress, which could be induced by amyloid β protein [34], [35] (Figures 2 and 6), 4: Senkyu inhibits the activation of caspase-4 under ER stress, which could lead to neural death [18], [19] (Figure 5), 5: Senkyu and Ferulic acid regulate the UPR signaling pathway, which is activated by amyloid β protein [36] (Figures 4 and 7), 6: Ferulic acid prevents cell death due to amyloid β protein toxicity [27] and also induces resistance to amyloid β1-42 toxicity in the brain [26]; Senkyu or ferulic acid alone may be suitable drugs for AD therapy because both medicines inhibit ER stress following amyloid β toxicity [7], [8], [18], [19], [34]–[36].

At present, the therapeutic drugs available for AD include cholinesterase inhibitors and NMDA-receptor antagonists. However, their therapeutic effect is not significant [37]–[40]. A number of trials to develop effective drugs for AD have been performed based upon the amyloid β hypothesis or tau hypothesis [41]–[46]. However, the development of a truly effective treatment for AD is far away.

It has been reported that neuronal death observed in AD is related to ER stress [4]–[12], [18], [19]. In this study, we used TG as an ER stressor. TG, a highly lipophilic sesquiterpene lactone, is broadly used as a selective inhibitor of sarcoplasmic reticulum calcium-ATPase (SERCA), which pumps calcium from the cytosol into the lumen of the ER in mammalian cells. TG-mediated irreversible inhibition of ER calcium-ATPases can also cause the induction of calcium leakage from the ER to the cytoplasm, further facilitating the depletion of calcium within the ER, resulting in an increase in cytoplasmic calcium levels [47]. Long-term elevation of intracellular calcium can induce ER stress due to misfolded protein accumulation [48], [49]. Our results show that Senkyu and Yokukansan were protective against TG toxicity (Figures 1, 2, 3 and 6). This study has shown that ferulic acid, Senkyu and Yokukansan could be potential drugs for the treatment of AD and sheds some light for the development of new AD therapies.

## Materials and Methods

### Yokukansan, its components and Senkyu-free Yokukansan

Yokukansan (TJ-54) consists of Sojutsu (Atractylodes Lancea rhizome), Bukuyo (Hoelen), Senkyu (Cnidii Rizoma), Chotoko (Uncariae Uncis Cum Ramulus), Toki (Angelicae Radix), Saiko (Bupleuri Radix) and Kanzo (Glycyrrhizae Radix). Yokukansan is extracted from a mixture of dried plants as follows; 4 g of Sojutsu, 4 g of Bukuryo, 3 g of Senkyu, 3 g of Toki, 2 g of Saiko, 1.5 g of Kanzo and 3 g of Chotoko were added to 700 ml of distilled water and boiled for 1 hour, filtered, and then concentrated to 300 ml. On the other hand, to prepare Senkyu-free Yokukansan, the same extraction-method and amount of Soujutsu, Bukuyo, Chotoko, Toki, Saiko and Kanzo were used. Yokukansan, components of Yokukansan and Senkyu-free Yokukansan were kindly provided by Tsumura & Co. (Tokyo, Japan).

### Chemicals and antibodies

We used the following antibodies: anti–Bip mAb (Cell Signaling Technology, Beverly, MA), anti-caspase-4/TX mAb (4B9; MBL International Corporation, Nagoya, Japan), monoclonal anti-β actin antibody (Chemicon, Temecula, CA) and HRP-conjugated anti–mouse IgG antibody (Cell Signaling Technology). The chemical reagents used in this experiment were thapsigargin (TG), staurosporine (STS) (Sigma-Aldrich, St. Louis, Mo) and ferulic acid (LKT Laboratories, Inc., St. Paul, MN).

### Cell Culture

SK-N-SH human neuroblastoma cells were obtained from the Riken Cell Bank (Tsukuba, Japan). Neuro-2a mouse neuroblastoma cells (N_2_a cells) were obtained from ATCC (Manassas, VA). Human neuroblastoma SK-N-SH cells and Mouse neuroblastoma N_2_a cells were cultured in DMEM (Sigma) containing 10% (v/v) fetal bovine serum and incubated in a humidified chamber at 37°C with a 5% CO_2_ atmosphere according to previous experiments [7]–[12]. SK-N-SH neuroblastoma cell lines stably expressing wild-type PS1 (PS1 WT cells) or PS1ΔE9 (PS1ΔE9 cells), which have been described previously [7], were cultured similarly to SK-N-SH cells.

### Cell viability assay based on morphological changes

Cell toxicity in N_2_a cells was measured on the basis of morphological changes observed by phase contrast microscopy or nuclear changes detected by fluorescence microscopy after co-staining cells with 10 µM Hoechst 33342 and 10 µM propidium iodide (PI). Hence, nuclear fragmentation was detected by Hoechst-positive staining and nuclear collapse was detected by PI-positive staining. Double positive cells were considered dead cells. Staining was measured independently in 4 fields and at least 300 cells were counted. Data are expressed as the mean ± SEM for at least three independent experiments.

### Cell viability assay by WST-1 activity

SK-N-SH cells, PS1 WT cells, or PS1ΔE9 cells (3×10^3^) were plated onto 96-well plates 36 h before cell viability was determined. Prior to performing the assay, cells were pretreated for 1.5 h with Yokukansan, or with every component of Yokukansan, at the indicated concentrations followed by the addition of each insult (TG or STS) for 3 h. Following insult exposure, cells were washed twice with phosphate buffered saline (PBS) and cultured with DMEM (D1145, SIGMA) and WST-1 mixed medium for 3 hours. WST-1 was measured at an absorption of λ450 nm – λ650 nm. Data are expressed as the mean ± SEM for at least three independent experiments.

### Western blot analysis

Treated cells were washed twice with PBS, harvested and lysed in TNE buffer (10 mM Tris-HCl, pH 7.8, 1 mM EDTA and 150 mM NaCl) containing 1% (v/v) NP-40 and protease inhibitor cocktail (Roche, Sydney, Australia). Equal amounts of protein were subjected to 12% (v/v) SDS-PAGE for GRP78/Bip, caspase-4 or β actin and transferred to PVDF membrane (Millipore, Bedford, MA). The membrane was blocked with 5% (w/v) skim milk and incubated with primary antibody, followed by incubation with an HRP-conjugated secondary antibody. Proteins were visualized with an ECL detection system (Amersham Biosciences, Piscataway, NJ).

### Exposure to hypoxia

For the hypoxic insult, cells were exposed to hypoxia for 6 h using an incubator equipped with a hypoxic chamber that maintained a humidified atmosphere with low oxygen tension (8 Torr) as described previously [9], [11], [12].

### RT-PCR

Total RNA was extracted from cultured SK-N-SH cells treated with the indicated reagents using the High Pure RNA Tissue Kit (Roche).The cDNA was synthesized from total RNA using the High Capacity cDNA Reverse Transcription Kit (Applied Biosystems). Oligonucleotide sequences used for PCR were as follows; For GRP78/Bip, forward: 5′-agcctggcgacaagagtg-3′, reverse: 5′-tccttgggcagtattggatt-3′; For CHOP, forward: 5′-gcgcatgaaggagaaagaac-3′, reverse: 5′-ccaattgttcatgcttggtg-3′; For GAPDH, forward: 5′-ccactcctccacctttgacg-3′, reverse; 5′-caccctgttgctgtagccaa-3′.

### Extraction of contents from Senkyu

Cnidii Rhizoma (Senkyu, 100 g) was powdered and extracted with hot water (500 mL, reflux, 1 h ×2). The water solution was concentrated under reduced pressure to obtain a water extract. The crude water-extract (100 mg) was dissolved in PBS (10 mL), and extracted for 10 min in an ultrasonic water bath. The extraction was repeated twice. The extracted solutions were combined and centrifuged at 10000 rpm for 10 min. The supernatant was recovered and diluted to the appropriate concentration with assay medium.

## Supporting Information

Figure S1 Screening of contents of Senkyu. Extracts of Senkyu were divided into #1-6 fractions. We preliminary checked the effect of each fraction (#1-6) against the neural toxicity of TG by using SK-N-SH cells. Fractions #1-5 did not show any protective effect under TG stimulation, but fraction #6 showed a protective effect against TG-induced ER stress.
(0.61 MB PDF)

Figure S2 List of contents contained in fraction #5 and #6
(0.68 MB EPS)

Figure S3 Cell toxicities of Yokukansan and its components. Cell toxicity of N2a cells was measured based on morphological changes. Cell death was measured 24 h after treatment with Yokukansan or each of the indicated components of Yokukansan (200 µg/ml). Non treated cells were used as a control. Quantitative data are expressed as the mean ± SEM for at least three independent experiments. The P value was compared with the control and calculated by Student's T test.
(0.68 MB EPS)
