# Supplementary figures and images for: Yokukansan Inhibits Neuronal Death during ER Stress by Regulating the Unfolded Protein Response
Source: PLoS One. 2010 Oct 12;5(10):e13280. doi: 10.1371/journal.pone.0013280 (PMC2953506; doi:10.1371/journal.pone.0013280)

**Figure S1**

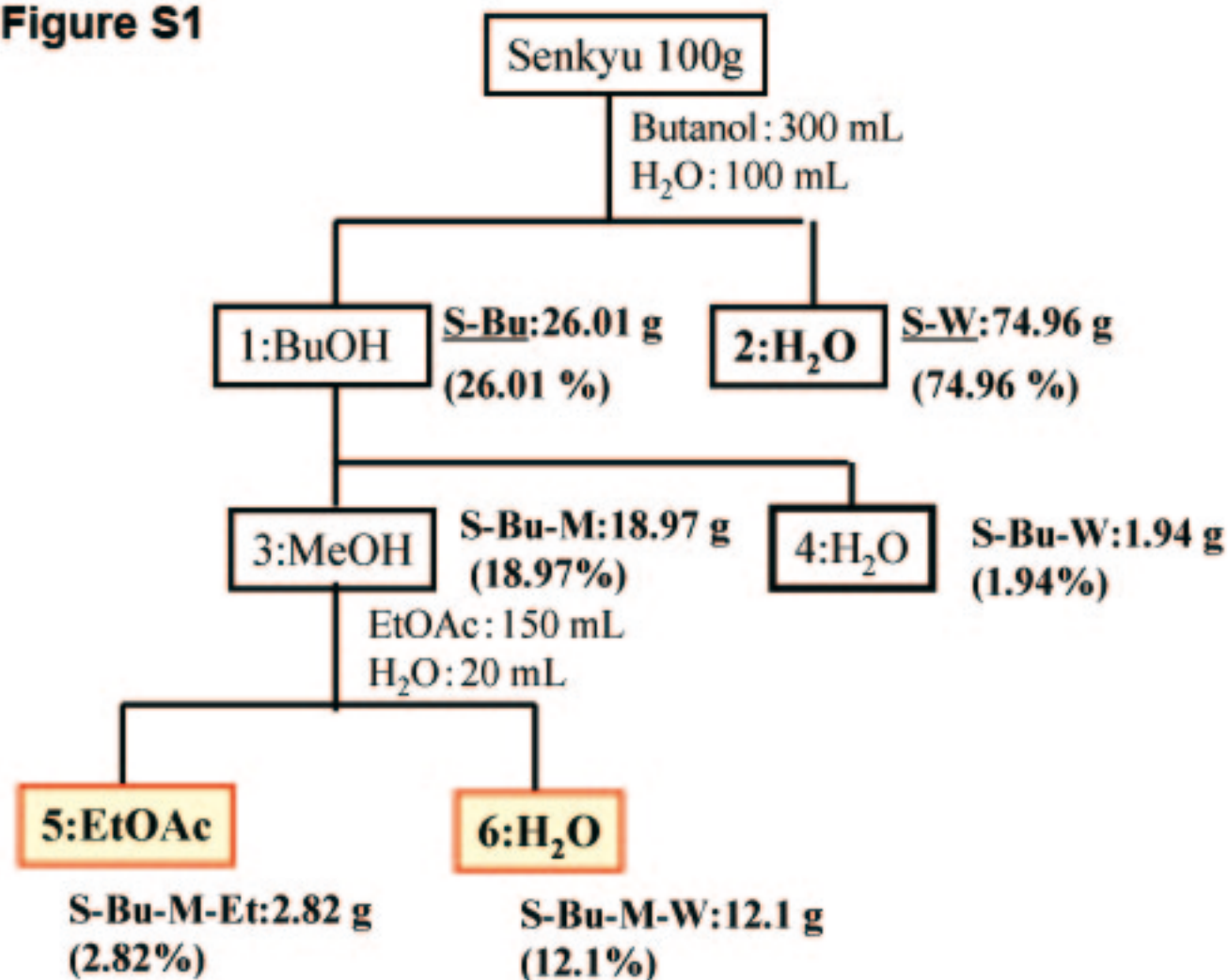

Supplement: Figure S1 — Screening of contents of Senkyu. Extracts of Senkyu were divided into #1-6 fractions. We preliminary checked the effect of each fraction (#1-6) against the neural toxicity of TG by using SK-N-SH cells. Fractions #1-5 did not show any protective effect under TG stimulation, but fraction #6 showed a protective effect against TG-induced ER stress. (0.61 MB PDF) [file pone.0013280.s001.pdf]
